# The Economics of Epidemic Diseases

**DOI:** 10.1371/journal.pone.0137964

**Published:** 2015-09-15

**Authors:** Nicola Dimitri

**Affiliations:** Department of Political Economy and Statistics, University of Siena, Siena, Italy; Universidad Veracruzana, MEXICO

## Abstract

Epidemic, infectious, diseases affect a large number of individuals across developing as well as developed countries. With reference to some very simple diffusion models, in this paper we consider how available economic resources could be optimally allocated by health authorities to mitigate, possibly eradicate, the disease. Optimality was defined as the minimization of the long run number of infected people. The main goal of the work has been to introduce a methodology for deciding if it would be best to concentrate resources to prevent contact between individuals and with an external source, or to develop a new treatment for curing the disease, or both. The analysis suggests that this depends on the cost functions, that is the available technology, for controlling the relevant parameters underlying the epidemics as well as on the available financial resources. In the case of the recent Ebola outbreak, the suggestions of the model have been consistent with the policies adopted.

## Introduction

Epidemic, contagious, diseases affect a large number of individuals across developing, as well as developed, countries. Though of different nature, such diseases share some common diffusion mechanisms ruling their dynamics and evolution within the relevant population. Since the eighteenth century [1],[2],[3],[4],[5],[6],[7] the more formal approaches to epidemic diseases clarified that there are four fundamental forces, common to different types of diseases, underlying the diffusion of an epidemics. (i) First, susceptible individuals can become infected by contact with a source that is external to the relevant population. For example, such source could be an animal carrying the infective agent, bacteria in water or in food, an infected individual coming from another population. (ii) The second main source of infection is contagion with an already infected individual of the same, relevant, community. This could happen for example by sexual contact, saliva and blood transmission. Finally, (iii) an infected person may be successfully treated, possibly if appropriate medication exists or, if treatment fails, (iv) the person may die. Spontaneous recovery can be seen as a special case of recovery with a zero-cost treatment.

At each date, the interaction of (i)-(iv) determines the number of susceptible individuals, that of infected people and of the infected who were cured and recovered as well as, in the worst cases of fatal diseases, the number of deaths.

If the fundamental forces driving diffusion mechanisms are currently well understood systematic investigation of the following related aspect, of comparable importance to fight such diseases, seems to be relatively recent [8],[9],[10]. Consider the outbreak of an infection and that the health authorities in charge are endowed with a budget *B* > 0 (say *euro*) of financial resources to fight, possibly defeat, the disease. How should the budget be optimally spent? More specifically, should the resources be focused on trying to discover a medical treatment, if not yet available, or should they be allocated to limit contact and infected people isolated to prevent transmission? Alternatively, should they be invested to eliminate the external source of infection, which would include prevention, or a combination of such actions?

The main goal of the paper is to introduce the issue and discuss some fundamental insights behind such decision, however with no ambition to propose a complete analysis of the several, different, epidemic models. The paper suggests how the “optimal” allocation of the financial resources depends on two main aspects: the nature of the diffusion mechanism and the cost structure for controlling transmission and removal. If this may not appear surprising perhaps less obvious, even in the simplest epidemic processes that we consider, would be the insights on how to best allocate the available resources to control the underlying driving forces.

## A Simplest Probabilistic Epidemic Model

To start identifying the main conceptual issues in this section we begin considering the simplest probabilistic epidemic model, where the initial population of susceptibles is composed by only one individual, threatened by a non-fatal disease. Despite its simplicity the model will convey most of the key insights of the approach, many of them extensible to a population of *N* individuals. Indeed, conclusions valid for a single person could rather simply be scaled up to *N* subjects. To study the evolution of the epidemics we introduce the time index *T* = 0,1,…, *t*,… The person is initially healthy but at time *T* = 0 an external source is carrying a, non-fatal, infective agent. Conditional to being healthy, the individual can be infected by the agent with probability 0 ≤ *α* ≤ 1 or remain healthy with probability 1 − *α*. If the person gets infected at *T* = 0 then, at *T* = 1, he could become healthy again with probability 0 ≤ *μ* ≤ 1, or remain infected with probability 1 − *μ*. Assume this diffusion mechanism to be valid at any date *T* and, still to simplify the exposition, that if the external source is not eliminated a treated individual could become infected again. Then the evolution of the epidemics can be summarized by Table 1 below, showing the transition probability matrix between the healthy and the infective state. Indeed, as we defined it, this model is the simplest example of a two-states (healthy-infected) Markov Chain.

**Table 1:** Transition probability matrix between the healthy and the infective state, in a one person population.

Number of Infected at	date *T* = *t* + 1	
date *T* = *t*	0	1
**0**	1 − *α*	*α*
**1**	*μ*	1 − *μ*

Suppose that to face the epidemics health authorities are endowed with a monetary budget *B* > 0 (*euro*) with which they would control the *infection*, *transmission*, probability (rate) *α* and the *removal* probability (rate) *μ*. The nature of the costs for controlling *α* and *μ* will determine how to best allocate the available budget. Hence if *C*(*α*) and *C*(*μ*) are, respectively, the cost functions for controlling the transmission and the removal (rate), then the budget constraint faced by the authority is given by *C*(*α*) + *C*(*μ*) ≤ *B*. Notice that while *C*(*μ*) is increasing in *μ* the cost function *C*(*α*) decreases with *α* Moreover, the functional form of the cost functions is very important since it reflects the state of knowledge, the available technology, concerning the disease. So a budget that for a rather well understood disease could be enough to enhance full control of the epidemics, it may be largely insufficient for a not-so-well understood disease.

In the simplest case, costs could be seen as proportional to the increase in the two rates as follows *C*(*α*) = *p*(1 − *α*) and *C*(*μ*) = *qμ*, where *p* and *q* are the non-negative costs for, respectively, complete elimination of the transmission, *α* = 0, and to obtain full removal, that is *μ* = 1. With no loss of generality, to further simplify the exposition, we normalize *p* = 1 so that the budget constraint becomes (1 − *α*) + *qμ* ≤ *B*. Indeed, if *p* is not initially equal to one then by dividing each term of the budget constraint by it *q* and *B* would now simply represent, respectively, the ratio between the cost of *μ* and that of (1 − *α*), and the maximum number of (1 − *α*) “units” that could be purchased with the available budget.

Once the infective agent is introduced, assuming the above diffusion mechanism to remain unaltered over time, health authorities should first have to understand how the epidemics evolves with time. In particular, after a sufficiently long period, what is the probability that the individual will finally be healthy or infected. Such long run probability (steady state) distribution is given by $\pi \left(0\right)=\frac{\mu }{\alpha +\mu }$ and $\pi \left(1\right)=\frac{\alpha }{\alpha +\mu }$ (see Supporting Information, S1 File).

Therefore, if *α* = *μ* then eventually it will be equally likely to have 0 or 1 infected individuals, while if *α* > *μ* the latter will be more likely, and the opposite if *α* < *μ*. If *X* is the number of people eventually infected, in this simplest diffusion model its expected value $EX=0\left(\frac{\mu }{\alpha +\mu }\right)+1\left(\frac{\alpha }{\alpha +\mu }\right)=\frac{\alpha }{\alpha +\mu }$ coincides with *π*(1), while its variance $VX=\frac{\alpha }{\alpha +\mu }\left(1-\frac{\alpha }{\alpha +\mu }\right)$ clearly reaches a maximum when the two rates are equal.

Then, for the health authorities an obvious goal to pursue may be to allocate the available financial resources between *α* and *μ* to minimize *EX*, or equivalently maximize $\frac{\mu }{\alpha +\mu }$, with respect to *α* and *μ* given the budget constraint (1 − *α*) + *qμ* ≤ *B*.

The following considerations provide interesting, non-obvious, insights together with some more natural conclusions. Given the diffusion mechanism, the size of the budget and the nature of the costs will guide the decision. This is why the analysis will proceed by considering different budget levels (see S1 File).

i) Suppose first 1 ≤ *B* that is, the available budget is high enough to completely eliminate infection transmission, *α* = 0 and so *EX* = 0. Then the best allocation of the available resources would be to invest 1, of the *B* euros available, to fully prevent contact and the rest in “buying” *up to* $\mu =Min\left(1,\frac{B-1}{q}\right)$ units of the removal rate. Indeed, if the speed with which the epidemics is defeated, in case it takes place, is also of concern then the level of the removal rate should be as high as possible.
ii) Suppose now *q* ≤ *B* < 1, that is full control of transmission is not possible while complete removal is possible. Then, because of the nature of the diffusion mechanism, the infection could not be eliminated and in this case the available resources would be best allocated by investing *q* of the *B* euro in setting *μ* = 1 and the remaining *B* − *q* euro in controlling *α*, to minimize the expected number of infected individuals and speed up convergence to $EX=\;\frac{1-\left(B-q\right)}{2-\left(B-q\right)}$. This tends to $\frac{1}{2}$ as *q* gets close to *B* and to $\frac{1-B}{2-B}$ as *q* approaches to 0. Therefore, with “sufficiently cheap” removal rate and a budget close to 1, that is almost capable to “buy” full control of transmission, the expected number of infected individuals would approach zero. Finally, since $\frac{1-\left(B-q\right)}{2-\left(B-q\right)}$ decreases with *B* − *q* over its domain [0,1), the long run probability of one infected person will always be lower than $\frac{1}{2}$.
iii) If the above conclusions may not be surprising, less obvious will be the findings for *B* < *Min*(1,*q*), when neither transmission nor removal of the disease could be completely controlled. In this case, how should the budget be spent? Should it be distributed between the two rates, and if yes how, or should investments be prioritized? The answer is the latter, and the analysis suggests that priority should be given to removal as it would be optimal now to spend the entire budget in”buying” units of the removal rate, hence setting $\mu =\frac{B}{q},\;\alpha =1$, to increase as much as possible the chance of successful recovery from the illness. In this case $EX=\;\frac{q}{B+q}\geq \frac{1}{2}$. Table 2 summarizes the above considerations

**Table 2:** Optimal allocation of the financial budget B for an epidemics in a one-individual population.

	*α*	*μ*	$\pi \left(0\right)\;=\frac{\mu }{\alpha +\mu }$	$\pi \left(1\right)\;=\frac{\alpha }{\alpha +\mu }$
***B* ≥ 1**	0	$\frac{B-1}{q}$	1	0
***q* ≤ *B* < 1**	1 − (*B* − *q*)	1	$\frac{1}{2-\left(B-q\right)}>\frac{1}{2}$	$\frac{1-\left(B-q\right)}{2-\left(B-q\right)}<\frac{1}{2}$
***B* < *Min*(1,*q*)**	1	$\frac{B}{q}$	$\frac{B}{B+q}<\frac{1}{2}$	$\frac{q}{B+q}>\frac{1}{2}$

It is important to point out that the cost structure plays a crucial role for the optimal allocation of resources. Indeed, suppose for example that the cost function of *α* would still be *C*(*α*) = (1 − *α*) but that now *C*(*μ*) = *qμ* ^2^, namely quadratic in *μ*. This means that costs for improving removal increase with *μ* but no longer at a constant, rather at an increasing, rate. In this case the budget constraint becomes (1 − *α*) + *qμ* ^2^ ≤ *B*.

It is easy to verify that for *B* ≥ 1 a similar conclusion holds as it would be optimal to set *α* = 0 and $\mu =Min\left(1,\sqrt{\frac{B-1}{q}}\right)$. However, now for the remaining two cases with *B* < 1, removal may not be prioritized and some of the resources spent also to “buy” units of (1 − *α*). Indeed with *q* ≤ *B* < 1 it could be checked that if $0<\left(1-B\right)<Min\left(q,\frac{1}{2}\right)$, that is $Max\left(1-q,\frac{1}{2}\right)<B<1$, then it would be optimal to choose $\mu =\sqrt{\frac{1-B}{q}}$ and *α* = 2(1 − *B*), with the relevant probabilities now given by $\pi \left(0\right)=\frac{1}{1+2\sqrt{q\left(1-B\right)}}$ and $\pi \left(1\right)=\frac{2\sqrt{q\left(1-B\right)}}{1+2\sqrt{q\left(1-B\right)}}$. Hence it could be verified that $\pi \left(1\right)\leq \frac{1}{2}$ if $q\leq \frac{1}{4\left(1-B\right)}$, which is always true since $B\leq \frac{1}{4\left(1-B\right)}$. The intuition behind such optimal choice is simple. Since 0 ≤ *μ* ≤ 1 and the cost of complete removal is still *q*, then *qμ* ^2^ ≤ *qμ*, so that controlling removal is now less expensive and as a consequence some financial resources could also be employed to control transmission. Therefore, a different cost structure changes the optimal allocation of available resources and, in turn, the relevant probabilities of infection.

## A More General Epidemic Model

Consider now a population with two individuals, and so three states with which to describe the epidemics. That is, at any date either zero, one or two people could be infected. The epidemics is such that, in one step, at most one individual could be treated or one individual is infected. Hence in a single step it is impossible to treat two individuals, to go from zero to two infected individuals as well as to remain with one infected individual because the healthy person was infected and at the same time the other healed. Moreover, transmission by contagion with an already infected individual is precluded. Table 3 describes the related transition probability matrix.

**Table 3:** Transition probability matrix between the healthy and the infective states, in a two-individuals population with no internal contagion.

Number of Infected at	date *T* = *t* + 1		
date *T* = *t*	0	1	2
**0**	1 − *α*	*α*	0
**1**	*μ*	1 – *α* – *μ*	*α*
**2**	0	*μ*	1 − *μ*

Since 0 ≤ *α* + *μ* ≤ 1 unlike the previous process now the model captures an epidemics where, for example, full control of removal *μ* = 1 implies also full control of contact and transmission, *α* = 0, that is the infection dying out. Analogously, the maximum strength of transmission, *α* = 1, implies that no removal is possible and that the infection eventually will be completely diffused.

It is easy to see that in this case the long run, steady state, probability distribution is given by

$$\pi \left(0\right)=\frac{\mu^{2}}{\alpha^{2}+\mu \left(\alpha +\mu \right)},\;\pi \left(1\right)=\frac{\alpha \mu }{\alpha^{2}+\mu \left(\alpha +\mu \right)},\;\pi \left(2\right)=\frac{\alpha^{2}}{\alpha^{2}+\mu \left(\alpha +\mu \right)}$$ (1)

Therefore, if the relevant costs are still *C*(*α*) = (1 − *α*) and *C*(*μ*) = *qμ*, then the Health Authorities budget constraint remains (1 − *α*) + *qμ* ≤ *B*. Therefore, the expected number of infected people is (S1 File)

$$EX=0\frac{\mu^{2}}{\alpha^{2}+\mu \left(\alpha +\mu \right)}+\frac{\alpha \mu }{\alpha^{2}+\mu \left(\alpha +\mu \right)}+\frac{2\alpha^{2}}{\alpha^{2}+\mu \left(\alpha +\mu \right)}$$ (2)

and it can be verified that now also in this case the condition *B* ≥ 1, that is full control of the external source, is fundamental to eliminate the infection. More specifically, in this case to minimize *EX* with respect to *α* and *μ*, given that 0 ≤ *α* ≤ 1 − *μ* and (1 − *B*) + *qμ* ≤ *α*, it will be optimal to invest 1 of the available *B* euro in fully eliminating contact and the rest in increasing as much as possible the removal rate, to speed up the elimination of the epidemic. However if 1 > *B* things differ, depending upon *B* being larger (smaller) than *q*. Indeed, it can now be checked (see supplementary material) that it is optimal to allocate the available resources setting $\mu =\frac{B}{1+q}$ and $\alpha =1-\frac{B}{1+q}$. Intuitively, the difference with respect to the previous simpler model is that now *α* can affect more than one person, which justifies investing resources also on it. Therefore, it will be the value of *q* to determine how much to invest in eliminating contact as compared to increasing the removal rate. Finally, notice that since *α* + *μ* = 1 then $=\frac{\alpha +\alpha^{2}}{\alpha^{2}+1-\alpha }$.

We now further extend the model by considering an individual who could become infected in two ways: he can either get the infection from the external source with probability *α* or, if already infected, from the other individual with probability *β*. Therefore, in this case the possibility of transmission and diffusion of the infection is strengthened.

Table 4 describes the related transition probability matrix.

**Table 4:** Transition probability matrix between the healthy and the infective states, in a two-individuals population with internal contagion.

Number of Infected at	date *T* = *t* + 1		
date *T* = *t*	0	1	2
**0**	1 − *α*	*α*	0
**1**	*μ*	1− *α* − *β* − *μ*	*α* + *β*
**2**	0	*μ*	1 − *μ*

Since 0 ≤ *α* + *β* + *μ* ≤ 1 the model captures an epidemics where full control of removal *μ* = 1 implies complete control of contact and transmission, *α* = 0 = *β*, that is the infection dying out, if the budget constraint is satisfied. Analogously, the maximum strength of transmission, *α* = 1 or *β* = 1, implies that no removal is possible and that the infection will be eventually completely diffused.

It is easy to see that in this case the long run probability distribution is given by (see SM)

$$\pi \left(0\right)=\frac{\mu^{2}}{\alpha \left(\alpha +\beta \right)+\mu \left(\alpha +\mu \right)},\;\pi \left(1\right)=\frac{\alpha \mu }{\alpha \left(\alpha +\beta \right)+\mu \left(\alpha +\mu \right)},\;\pi \left(2\right)=\frac{\alpha \left(\alpha +\beta \right)}{\alpha \left(\alpha +\beta \right)+\mu \left(\alpha +\mu \right)}$$ (3)

Hence, if now the relevant cost functions are *C*(*α*) = 1 – *α*, *C*(*β*) = *p*(1 − *β*) and *C*(*μ*) = *qμ*, then the health authorities’ budget constraint becomes (1 − *α*) + *p*(1 − *β*)+*qμ* ≤ *B*. Therefore, the expected number of infected people is

$$EX=0\;\left(\frac{\mu^{2}}{\alpha \left(\alpha +\beta \right)+\mu \left(\alpha +\mu \right)}\right)+1\;\left(\frac{\alpha \mu }{\alpha \left(\alpha +\beta \right)+\mu \left(\alpha +\mu \right)}\right)+2\;\left(\frac{\alpha \left(\alpha +\beta \right)}{\alpha \left(\alpha +\beta \right)+\mu \left(\alpha +\mu \right)}\right)$$ (4)

which would be equal to zero for *μ* = 1 or *α* = 0.

Therefore, in this case the best allocation of resources obtains by minimizing *EX* with respect to *α*, *β* and *μ*, subject to the budget constraint and to 0 ≤ *α* + *β* + *μ* ≤ 1. Hence, if 1 < *B* then it is still optimal to spend 1 euro setting *α* = 0 = *EX*, and the remaining resources *B* − 1 in controlling the speed with which the epidemics spreads. However, if 1 > *B*, that is full control of the contact rate is too expensive, the question is where it is optimal to invest the available resources in order to minimize the expected number of infected individuals. The analysis suggests (see SM) that if *p* ≤ *B* < 1 then $\beta =0,\;\mu =\frac{B-p}{1+q}$ and $\alpha =1-\left(B-p\right)+\frac{q\left(B-p\right)}{1+q}=1-\frac{B-p}{1+q}$. That is, in this case it is optimal to eliminate completely transmission by contact between individuals and allocate the remaining resources *B* − *p* between *α* and *μ*. The relative costs of the two parameters will determine which of them will be larger.

## The Ebola Epidemics

Since the recent major outbreak of the Ebola virus (EV), formally reported for the first time by the World Health Organization (WHO) on 3 August 2015, there have been almost 11.290 deaths.

Until very recently, there was no licensed pharmaceutical treatment specific for EV [11], and strategies to fight the virus have been mostly based on trying to contain the epidemics. However, in a recent paper [12] Henao-Restrepo et al. (2015) report very encouraging results, of a wide experimentation in Guinea, on the efficacy and effectiveness of a vaccine for EV. The lack of a dedicated treatment was likely to be due to the sporadic and circumscribed previous outbreaks, which despite the seriousness of the disease so far were not enough to justify systematic research and development (R&D) effort to produce a drug or vaccine. The recent outbreak was very different from previous episodes and R&D effort went now at work. However, while hoping that an effective pharmaceutical treatment was to be soon available, recent proposals suggested that a policy combining the following main measures could help to limit the spreading of the disease: “case isolation, contact-tracing with quarantine, and sanitary funeral practices”[11]. Such suggestions seem consistent with those from our very simple model with internal contagion, as illustrated in Table 4. The absence of a specific, effective, treatment can be interpreted as due to low available financial resources, which should be increased in order to eradicate, or keep under close control, the disease. While R&D activity was supported, most of the resources available were employed to prevent contagion transmission between infected and healthy individuals, as indeed both the proposed policy measures and the model findings suggest.

Despite its simplicity and several limitations, such as for example assuming that the disease is not fatal, our model appears to be catching some main insights on effective resource allocation to fight an infectious epidemics.

## Conclusions

In the paper we considered three very simple dynamic, probabilistic, epidemic models to gain some broad insights on how health authorities could best allocate the available financial resources to fight an infectious disease. Though simple the models deliver interesting indications on how health authorities may proceed. The main point of the analysis is that the optimal allocation of such resources depends on the nature of the diffusion process and of the cost structure for controlling the relevant forces driving the epidemics. In particular, the simplest model with no contagion between infected individuals suggests that when resources are neither enough to fully control the external infective source of transmission, nor to completely cure the disease, they should all be spent in trying to increase as much as possible the removal rate. We do not think this to be an obvious indication as, perhaps, at first the most natural way to proceed might seem to be to invest all the available resources to try mitigating the impact of the external infective source. However, in the paper we also point out how these indications might be sensitive to the form of the cost functions and that changing them different conclusions, on how to best allocate available resources, could be reached. The more elaborated model, encompassing the possibility of contagion between infected individuals, is also providing useful insights. For the optimal allocation of resources, the following two cases are the most interesting ones. If it is too expensive to fully control the external source of infection as well as to find a treatment that would fully cure the disease, but only elimination of interpersonal contact is affordable, then it is best to fully control transmission between individuals and allocate the remaining resources to both trying to mitigate the strength of the external source of infection as well as to increase the removal rate.

As we elaborate in section 4, this last conclusion appears to be consistent with real life situations where, facing an epidemics, as a first policy measures Health Authorities in charge isolate infected individuals, separating them from the healthy ones. Indeed, separation is normally a cheap and wise enough precautionary measure to undertake, notably under realistic situations in which the cost functions are not precisely known or, even more so, when the underlying causes of the epidemics are not completely understood and/or a treatment is not yet available.

## Supporting Information

S1 File The probabilistic and deterministic models. (DOCX)
