# Supplementary material for: The Economics of Epidemic Diseases
Source: PLoS One. 2015 Sep 15;10(9):e0137964. doi: 10.1371/journal.pone.0137964 (PMC4570787; doi:10.1371/journal.pone.0137964)
Supplement: S1 File — (DOCX) [file pone.0137964.s001.docx]

$\pi\left( 0 \right)=\frac{\mu^{2}}{\alpha^{2}+\mu(\alpha+\mu)}$ $\pi\left( 0 \right)=\frac{\mu^{2}}{\alpha\left( \alpha+\beta\right)+\mu(\alpha+\mu)}$

$$EX=0\left( \frac{\mu^{2}}{\alpha\left( \alpha+\beta\right)+\mu\left( \alpha+\mu\right)} \right)+1\left( \frac{\alpha\mu}{\alpha\left( \alpha+\beta\right)+\mu\left( \alpha+\mu\right)} \right)+2\left( \frac{\alpha(\alpha+\beta)}{\alpha\left( \alpha+\beta\right)+\mu\left( \alpha+\mu\right)} \right) (4)$$

**S1 File**

**1 The probabilistic models**

In this section we discuss how conclusions of the main text obtain. The three simple models considered are all Markov Chains, that is stochastic processes where at each date $T+1$, for any $T=0,1,2,..,$ the number of infected individuals depends, probabilistically, only on the number of infected people at $T$, and not on previous dates. Hence, if $s=0,1,2,..,S$ is the generic state of the system (that is the number of infected individuals), with $S$ being the number of states, and $\pi\left( T \right)=\left( \pi_{T}\left( 0 \right),\ldots,\pi_{T}\left( S \right) \right)$ the $S-$dimensional probability vector for the system to be at state $s$ at time $T$, then the evolution of the system is fully described by the following simple dynamic difference equation

$$\pi\left( T+1 \right)=\pi\left( T \right)\Pi(5)$$

where $\Pi$ is the $S*S$ stationary transition probability matrix of the system. In particular, its generic element $\pi(i,j)$ gives the probability of being at state $j=0,1,..,S$ at time $T+1$ conditional to being at state $i=0,1,..,S$ at time $T$. Hence, by recursive substitution it is possible to express $\pi(T)$ as follows

$$\pi\left( T \right)=\pi\left( 0 \right)\Pi^{T}(6)$$

As $T$ gets large, under appropriate conditions on the transition matrix, the probability vector tends to a limit $\pi$ regardless of the initial condition $\pi(0)$. This vector defines the so called steady state (long run) probability distribution, which can be found solving in $\pi$ the following system of equations

$$\pi=\pi\Pi(7)$$

that is, the long run distribution is independent of $T.$

Based on this, since for the model in Table 1 the transition matrix of the system is

$$\Pi=\left[ \begin{matrix} 1-\alpha& \alpha\\ \mu& 1-\mu\end{matrix} \right]$$

then the long run probability distribution solves the system of equations

$$\pi\left( 0 \right)\left( 1-\alpha\right)+\pi\left( 1 \right)\mu=\pi\left( 0 \right)$$

$$\pi\left( 0 \right)\alpha+\pi\left( 1 \right)(1-\mu)=\pi\left( 1 \right)$$

and because $\pi\left( 0 \right)=1-\pi\left( 1 \right)$ it follows that

$\pi\left( 0 \right)=\frac{\mu}{\alpha+\mu}$ and $\pi\left( 1 \right)=\frac{\alpha}{\alpha+\mu} (8)$

Indeed since, with $0<\alpha,\mu<1,$ the eigenvalues of $\Pi$ are given by $1$ and $1-\alpha-\mu$ it is well known that that

$$\Pi^{T}=\left[ \begin{matrix} \frac{\mu}{\alpha+\mu}+\frac{\alpha}{\alpha+\mu}{(1-\alpha-\mu)}^{T} & \frac{\alpha}{\alpha+\mu}-\frac{\alpha}{\alpha+\mu}{(1-\alpha-\mu)}^{T} \\ \frac{\mu}{\alpha+\mu}-\frac{\mu}{\alpha+\mu}{(1-\alpha-\mu)}^{T} & \frac{\alpha}{\alpha+\mu}+\frac{\mu}{\alpha+\mu}{(1-\alpha-\mu)}^{T} \end{matrix} \right]$$

whose columns converge exponentially to the steady state probability distribution.

Therefore, the goal of the health authority is to minimize the expected number of infected individuals

$$EX=0\left( \frac{\mu}{\alpha+\mu} \right)+1\left( \frac{\alpha}{\alpha+\mu} \right)=\frac{\alpha}{\alpha+\mu} (9)$$

with respect to $\alpha$ and $\mu,$ given the budget constraint $\left( 1-\alpha\right)+q\mu\leq B.$ To solve such problem simply fix $\frac{\alpha}{\alpha+\mu}=k$ to obtain $\alpha=\frac{k}{(1-k)}\mu$ and identify the set of pairs $(\mu,$ $\alpha)$ providing the same expected number $EX=k$ of people eventually infected. Hence, in the unit square domain of $(\mu,$ $\alpha)$, this is a straight line stemming from the origin, with slope $\frac{k}{(1-k)}.$ Therefore, minimizing $EX$ amounts to minimizing the slope $\frac{k}{(1-k)}$ with respect to $k$, subject to $\left( 1-\alpha\right)+q\mu\leq B$, an area which is defined with reference to the straight, budget, line $\left( 1-B \right)+q\mu$ as follows $\left( 1-B \right)+q\mu\leq\alpha$, in the space $(\mu,$ $\alpha).$ The budget line $\left( 1-B \right)+q\mu$ would of course have intercept $\left( 1-B \right)$ and slope $q.$

Thus, if $B\geq1$ then $\left( 1-B \right)\leq0$ and the vertical intercept of the constraint is non-positive. As a consequence, it is easy to see that if $B\geq1+q,$ then resources are enough to fully control both transmission and removal $(\mu=1,$ $\alpha=0)$, thus $EX=0$ since from the budget line computed at $\mu=1$, that is $\left( 1-B \right)+q$, it is $\alpha\leq0$. However, if $1\leq B<1+q$ then at $\mu=1$ the value of $\alpha$ given by the budget line is positive and so we could still obtain full elimination of the epidemics, $\alpha=0$ and $EX=k=0$, however now with maximum removal rate $\mu=\frac{B-1}{q}$, that is equal to the largest number of units of $\mu$ that could be “bought” after having invested $1$, of the $B$ available euro, in eliminating contact. As said in the main text, the value of $\mu$ will drive the speed with which the infection, if it ever takes place, will eventually die out. The intuition is simple: once transmission from an external source is eliminated then the higher the removal rate the sooner the infection will be defeated.

However, if $B<1$ then the vertical intercept of the budget line is positive and $EX=k>0$ since financial resources are not enough to fully control infection transmission. In this case, again, to minimize $EX=k$ we need to check the value of the budget line at $\mu=1$. If $\left( 1-B \right)+q\leq1$, that is $q\leq B$ then removal could be complete, and it is simple to see that it would be optimal to invest $q$, of the available $B$ euro, to set $\mu=1$ and the rest to have $\alpha=1-(B-q)$. Hence the epidemics cannot be completely defeated but the average number of infected people will be no higher than a half. Finally, if $B<q$ then the budget line will take a value higher than one at $\mu=1$ and $EX=k$ could be minimized by spending all the available resources to increase as much as possible the removal rate up to $\mu=\frac{B}{q}$, completely disregarding control of transmission, hence leaving $\alpha=1$. In this case, because costs are relatively high with respect to the budget, the expected number of infected people will be larger than a half.

In the main text of the paper we pointed out that the linearity of the costs plays an important role in the optimal resource allocation. Indeed with a quadratic, rather than linear, cost function for the removal rate $\left( 1-\alpha\right)+q\mu^{2}$, if $q\leq B<1$ and $0<\left( 1-B \right)\leq Min(q,\frac{1}{2})$, namely $Max(1-q,\frac{1}{2})\leq B<1$, then the optimal allocation of resources could be found at the tangency between the line $\alpha=\frac{k}{(1-k)}\mu$ and the budget curve $\alpha=\left( 1-B \right)+q\mu^{2}$, given by the first order condition $2q\mu=\frac{k}{(1-k)}=\frac{\alpha}{\mu}$, from which $\mu=\sqrt{\frac{1-B}{q}}$and $\alpha=2(1-B)$. The above conditions on $B$ guarantee that both parameters are no larger than $1$.

Considering now the model in Table 2, the transition probability would be

$$\Pi=\left[ \begin{matrix} 1-\alpha& \alpha& 0 \\ \mu& (1-\mu-\alpha) & \alpha\\ 0 & \mu& (1-\mu) \end{matrix} \right]$$

with the long probability distribution now solving the system of equations

$$\pi\left( 0 \right)\left( 1-\alpha\right)+\pi\left( 1 \right)\mu=\pi\left( 0 \right)$$

$$\pi\left( 0 \right)\alpha+\pi\left( 1 \right)\left( 1-\mu-\alpha\right)+\pi\left( 2 \right)\mu=\pi\left( 1 \right)$$

$$\pi\left( 1 \right)\alpha+\pi\left( 2 \right)(1-\mu)=\pi\left( 2 \right)$$

Hence, since $\pi\left( 2 \right)=1-\pi\left( 1 \right)-\pi\left( 0 \right)$ the steady state probability distribution is given by

$\pi\left( 0 \right)=\frac{\mu^{2}}{\alpha^{2}+\mu(\alpha+\mu)}$, $\pi\left( 1 \right)=\frac{\alpha\mu}{\alpha^{2}+\mu(\alpha+\mu)}$, $\pi\left( 2 \right)=\frac{\alpha^{2}}{\alpha^{2}+\mu(\alpha+\mu)} (10)$

In this case the optimal resource allocation would minimize

$$EX=\frac{{\alpha\mu+2\alpha}^{2}}{\alpha^{2}+\mu(\alpha+\mu)} (11)$$

with respect to $\alpha$ and $\mu$, given the constraints $\alpha\leq1-\mu$ and $\left( 1-B \right)+q\mu\leq\alpha.$ Following the same procedure as before we can pose

$$0\leq EX=\frac{{\alpha\mu+2\alpha}^{2}}{\alpha^{2}+\mu\left( \alpha+\mu\right)}=k\leq2 (12)$$

to determine the $(\mu,\alpha)$ pairs providing the same expected number of infected people. For $0<k<2$, expressing $\alpha$ as a function of $\mu$ we obtain

$$\alpha=\frac{\mu\left[ \sqrt{\left( 1-k \right)^{2}+4k\left( 2-k \right)}-\left( 1-k \right) \right]}{2(2-k)} \left( 13 \right)$$

from which $\frac{d\alpha}{d\mu}=\frac{\left[ \sqrt{\left( 1-k \right)^{2}+4k\left( 2-k \right)}-\left( 1-k \right) \right]}{2(2-k)}>0.$ If instead $k=0$ then $\alpha=0$ and $\mu>0$ while if $k=2$ then $\mu=0$ and $\alpha>0$. Therefore, in general, the same expected number of infected individuals can be obtained with $\mu$ and $\alpha$ varying according to the linear relationship $(13)$, stemming from the origin. Moreover, it is

$$\frac{dEX}{d\alpha}=\frac{dk}{d\alpha}=\frac{\mu(\mu^{2}+\alpha^{2}+4\alpha\mu)}{{{[\alpha}^{2}+\mu(\alpha+\mu)]}^{2}}>0 (14)$$

implying that, for given $\mu$, the expected number of infected individuals increases with $\alpha$.

Therefore, also in this case the analysis of the optimal resource allocation proceeds considering different levels of the budget. Indeed, if $B\geq1+q$ then resources are enough to completely control transmission, $\alpha=0,$ and removal $\mu=1.$If $1<B<1+q$ the solution is as in the previous model given by $\alpha=0$ and $\mu=\frac{B-1}{q}$. In all other cases, it is possible to verify that the right most point of the set, in the parameter space $(\mu,\alpha)$, defined by the constraints is always an optimal solution. That is the point where the two constraints meet, which solves

$1-\mu=\left( 1-B \right)+q\mu(15)$

hence $\mu=\frac{B}{1+q}$ and $\alpha=1-\frac{B}{1+q}$.

Finally, as for the model in Table 3 is concerned, the transition probability matrix is given by

$$\Pi=\left[ \begin{matrix} 1-\alpha& \alpha& 0 \\ \mu& (1-\mu-\alpha-\beta) & (\alpha+\beta) \\ 0 & \mu& (1-\mu) \end{matrix} \right]$$

with the stationary probability distribution now solving the system of equations

$$\pi\left( 0 \right)\left( 1-\alpha\right)+\pi\left( 1 \right)\mu=\pi\left( 0 \right)$$

$$\pi\left( 0 \right)\alpha+\pi\left( 1 \right)\left( 1-\mu-\alpha-\beta\right)+\pi\left( 2 \right)\mu=\pi\left( 1 \right)$$

$$\pi\left( 1 \right)(\alpha+\beta)+\pi\left( 2 \right)(1-\mu)=\pi\left( 2 \right)$$

Hence, the steady state probability distribution is given by

$\pi\left( 0 \right)=\frac{\mu^{2}}{\alpha\left( \alpha+\beta\right)+\mu(\alpha+\mu)}$, $\pi\left( 1 \right)=\frac{\alpha\mu}{\alpha\left( \alpha+\beta\right)+\mu(\alpha+\mu)}$, $\pi\left( 2 \right)=\frac{\alpha\left( \alpha+\beta\right)}{\alpha\left( \alpha+\beta\right)+\mu(\alpha+\mu)} (16)$

The model extends the previous one in the sense of still having three states, however now with three parameters rather than two. In this case the optimal resource allocation would minimize

$$EX=\frac{\alpha\mu+2\alpha(\alpha+\beta)}{\alpha(\alpha+\beta)+\mu(\alpha+\mu)} (17)$$

with respect to $\alpha$,$\beta$ and $\mu$ given the constraints $\alpha\leq1-\mu-\beta$ and $\left( 1-B \right)+\left( 1-\beta\right)p+q\mu\leq\alpha$. Following a similar procedure as before we can first consider the optimal choice for the pair of parameters $(\alpha,\beta)$ keeping $\mu$ as fixed, and then select $\mu$ optimally. To do so we pose again

$$EX=\frac{\alpha\mu+2\alpha(\alpha+\beta)}{\alpha(\alpha+\beta)+\mu(\alpha+\mu)}=k (18)$$

to determine the $(\alpha,\beta)$ pairs providing the same expected number of infected people. For $0<k<2$, expressing $\alpha$ in terms of $\mu$ we obtain

$$\alpha=\frac{\sqrt{{[\left( 2-k \right)\beta+\mu\left( 1-k \right)]}^{2}+4k\mu^{2}\left( 2-k \right)}-[\left( 2-k \right)\beta+\mu\left( 1-k \right)]}{2(2-k)} \left( 19 \right)$$

from which $\frac{d\alpha}{d\beta}<0$ if $-\left( 2-k \right)+\frac{\left[ \left( 2-k \right)\beta+\mu\left( 1-k \right) \right]\left( 2-k \right)}{\sqrt{\left[ \left( 2-k \right)\beta+\mu\left( 1-k \right) \right]^{2}+4k\mu^{2}\left( 2-k \right)}}<0$, that is $\frac{\left[ \left( 2-k \right)\beta+\mu\left( 1-k \right) \right]}{\sqrt{\left[ \left( 2-k \right)\beta+\mu\left( 1-k \right) \right]^{2}+4k\mu^{2}\left( 2-k \right)}}<1$ which is true. This means that, for given $\mu,$ to keep constant the expected number of infected individuals the transmission rate from an external source, and the contact rate between members of the population, must be negatively related. Notice also that at $\beta=0$ expression $\left( 19 \right)$ becomes $\left( 13 \right)$; that is, in the plane $(\alpha,\beta)$expression $(13)$ represents the intercept on the $\alpha$ axis which, as above, increases with $k$.

If instead, $k=0$ then $\alpha=0$ and $\mu>0$ while if $k=2$ then $\mu=0$and $\alpha>0$. Moreover, notice that

$$\frac{dEX}{d\mu}=\frac{dk}{d\mu}=-\frac{\left( \alpha^{3}+4\alpha^{2}\mu+\alpha\mu^{2}+4\alpha\beta\mu+\alpha^{2}\beta\right)}{\left[ \alpha\left( \alpha+\beta\right)+\mu\left( \alpha+\mu\right) \right]^{2}}<0 (20)$$

that is the expected number of infected people decreases as the removal rate increases.

Also in this case the optimal resource allocation depends on the available budget and the cost functions. In particular, if $B\geq1$ then the external source could be completely eliminated and the expected number of infected reduced to zero. With $B=1$ there would be no resources left to fight the epidemics in case it already took place. That is, the above conclusion holds only if the epidemics did not diffuse yet, so that there is no removal since there is no epidemics to stop. With $B>1$ instead there would be resources available to remove the infection with positive probability, in case it took place. Such positive probability of removal is enough for the epidemics to die out eventually. If instead $B<1$ the resources are not sufficient to eliminate the infection.

In this case it is easy to check that the budget constraint $\left( 1-B \right)+\left( 1-\beta\right)p+q\mu\leq\alpha$ is a subset of the positive unit square, where pairs $(\alpha,\beta)$ are defined. Therefore, solutions will exist as long as $\left( 1-B \right)+\left( 1-\beta\right)p+q\mu\leq1-\beta-\mu$ that is if $\mu\leq\frac{B-p}{1+q}$, which could be satisfied only if $B\geq p.$ Therefore now, since $\frac{dEX}{d\mu}<0, \frac{d\alpha}{d\beta}<0$and because at $\beta=0$ it is $\frac{d\alpha}{dk}<0$ it would be optimal to set the removal rate at its highest value, $\mu=\frac{B-p}{1+q}$, which implies $\beta=0$ and $\alpha=1-\frac{B-p}{1+q}$.

**2 A deterministic model**

In this section we discuss the optimal resource allocation in a simplest version of the well-known deterministic epidemic SIS (susceptible, infected, susceptible) model, a framework based on the “Law of Mass Action”[1]. The law formalizes the idea that if people in a population mix homogeneously, then the number of newly infected individuals is proportional to the number of possible contacts between the two groups (healthy-infected), that is to the product of the number of people in the two groups. Hence, in such model the evolution of the infection has no reference to probabilities. This is a classic approach to diffusion processes, although homogeneous mixing has recently been challenged by the network approach to the theory of epidemics [2],[3],[4],[5],[6].

In what follows $n(t)$ represents the number of people infected at date $t$, in a community of $N$ subjects, with $n\left( 0 \right)>0$ being the number of individuals initially infected, possibly by an external source, which for simplicity of exposition we do not formalize in the model. Moreover, $n=n(\beta$,$\mu)$ indicates the number of people eventually infected, that is after a long enough period of time, where $0\leq\beta,\mu\leq1$ are, respectively, the removal and the transmission (infection) rate. In the simplest version of the model the evolution of the epidemics is ruled by the following differential equation

$$\frac{dn\left( t \right)}{dt}=\beta n\left( t \right)\left[ N-n\left( t \right) \right]-\mu n\left( t \right) (21)$$

formalizing the idea that in the infinitesimal time interval, between dates $t$ and $t+dt$, the number of infected individuals increases proportionally to the product between the number of infected and the number of susceptibles, and decreases proportionally to those infected.

The problem, in a simplest setting, can be formulated as follows

$${Min}_{\beta,\mu}n\left( \beta,\mu\right)\geq0 (22)$$

such that $n(\beta,\mu)$ solves the equation

$$\frac{dn\left( t \right)}{dt}=\beta n\left( t \right)\left[ N-n\left( t \right) \right]-\mu n\left( t \right)=0 (23)$$

given the budget constraint

$$\left( 1-\beta\right)+\mu q\leq B (24)$$

where $q$ is the amount of money needed to “buy”, and fully control, the removal rate $\mu$ (that is $\mu=1)$ and, again, for simplicity $1$ is the price to “buy” and fully control the transmission rate (namely $\beta=0)$while $B$ is the available financial budget. Equation (23) is analogous to that for computing the steady state probability distribution of the previous probabilistic model, where now stationarity translates into the rate of growth of infected people being zero. Though both (23) and (24) can be made much more articulated, they are already rich enough for our, preliminary, analysis.

It is easy to check that the non-zero solution of (23) is given by $0\leq n=N-\frac{\mu}{\beta}\leq N$. That is, once the disease has spread the final number of infected people depends upon $\rho=\frac{\mu}{\beta}\geq0$, namely on the strength of the removal, as compared to the transmission, rate. The higher $\rho$ the lower the number of people eventually infected. Indeed, if $\rho<1$, that is $\beta>\mu$, the epidemics will eventually hit the entire population.

How should $B$ be allocated between $\mu$ and $\beta$ to best control the spreading of the disease?

Whenever possible, it would be optimal to select $\mu$ and $\beta$ in such a way that $n=0$, that is $\rho=N$. But as we shall see, also in this model, depending on the available budget and the costs this may not always be possible. In fact, by looking at the diffusion mechanism we can have a first intuition on how to proceed. If the budget is large enough to fully control the transmission rate (that is $B>1)$then the epidemics will eventually die out. Of course, since $n\left( 0 \right)>0$, for this to occur it cannot be $\mu=0.$

However, if the budget does not allow full control of the infection rate it is not obvious how the available resources should be allocated between the two rates. That is, should they be distributed between them or concentrated only on one?

More in general, from (23) we obtain that in the $(\beta,\mu)$ space the following linear equation expresses how the two rates must be connected, for any given number $n$ of eventually infected people

$$\mu=\beta\left( N-n \right) (25)$$

Still in the $(\beta,\mu)$ space, inequality (24) can be expressed as

$$\mu\leq\frac{B-1}{q}+\frac{\beta}{q} (26)$$

Hence, it is immediate to check that if $B-1<0$ and $q>B$ then the optimal solution is to choose $\beta=1$ and $\mu=\frac{B}{q}$, from which $n=N-\frac{B}{q}\sim N$. This means that all the available financial resources should be invested in the removal rate, with no attempt to reduce the transmission rate. Yet, since $\frac{B}{q}<1$ this could not preclude the epidemics from hitting the entire population, because finding a successful treatment is too expensive. However, if $q\leq B$ then resources allocation changes and becomes affordable, as well as optimal, to choose $\mu=1$ and $\beta=q+\left( 1-B \right)$. Therefore, $n=N-\frac{1}{q+(1-B)}\geq0$ implies $q+(1-B)\geq\frac{1}{N}$.

**References**

1 Daley DJ., Gani J., Epidemic modeling, Cam. Uni. Press, 1999

2 Keeling M, Earnes KTD, Networks and epidemic models, Jour. Roy Soc. Interf. 2005, 2,295-307

3 Easly D. Kleinberg J., Networks, crowds and markets: reasoning about a highly connected world. Cam. Uni. Pre. 2010

4 Balcan D. Colizza V., Goncalves B., Hu H., Ramasco JJ., Vespignani A., Multiscale mobility networks and the large scale spreading of infectious diseases, PNAS, 2005, 106, 21484-21489,

5 Colizza V., Vespignani A., The flu fighters, Phys World, 2010, 23, 26-30,

6 Casti J, X-events: the collapse of everything, Harper and Collins, 2012
